# Promotion of liquid-to-solid phase transition of cGAS by Baicalein suppresses lung tumorigenesis

**DOI:** 10.1038/s41392-023-01326-6

**Published:** 2023-03-22

**Authors:** Tiansheng Zheng, Haipeng Liu, Yifan Hong, Yajuan Cao, Qing Xia, Chengge Qin, Ming Li, Russel J. Reiter, Yidong Bai, Lihong Fan

**Affiliations:** 1 Department of Respiratory Medicine, Shanghai Tenth People’s Hospital, Tongji University School of Medicine, Shanghai, 200072 China; 2 Department of Integrated Traditional Chinese & Western Medicine, Shanghai Tenth People’s Hospital, Tongji University School of Medicine, Shanghai, 200072 China; 3 Clinical Translational Research Center, Shanghai Pulmonary Hospital, Tongji University School of Medicine, Shanghai, 200433 China; 4 Institute of Energy Metabolism and Health, Shanghai Tenth People’s Hospital, Tongji University School of Medicine, Shanghai, 200072 China; 5 Institute of Molecular Physiology, Institue of Cancer Research, Shenzhen Bay Laboratory, Shenzhen, 518132 China; 6 Medical School of Nantong University, Nantong, Jiangsu 22601 China; 7 Department of Cell Systems and Anatomy, University of Texas Health San Antonio, San Antonio, Texas 78229 USA

**Keywords:** Drug development, Lung cancer

**Dear Editor**,

Kras and p53 mutation are among the most common gene mutations in lung cancer, which has both the highest incidence and mortality rate among cancers.^1^ Kras/p53 mutation also causes mitochondrial dysfunction, which has been implicated to promote the inflammation-to-cancer transition.^2^ We established a lung adenocarcinoma model by using conditional alleles of Kras^LSL-G12D^/p53^flox/flox^ in mice^3^ to evaluate the effect of Baicalein (5,6,7-trihydroxyflavone), a principal component of *Scutellaria baicalensis* in traditional Chinese medicine,^4^ on the initiation and progression of lung cancer. Cre-mediated expression of Kras^G12D^ and deletion of p53 caused obvious tumor lesions in the lung, which were strongly inhibited by the administration of Baicalein (Fig. 1a, b and Supplementary Fig. 1a, b), indicating that Baicalein is highly potent in inhibiting the progression of primary lung cancer.

**Fig. 1:**
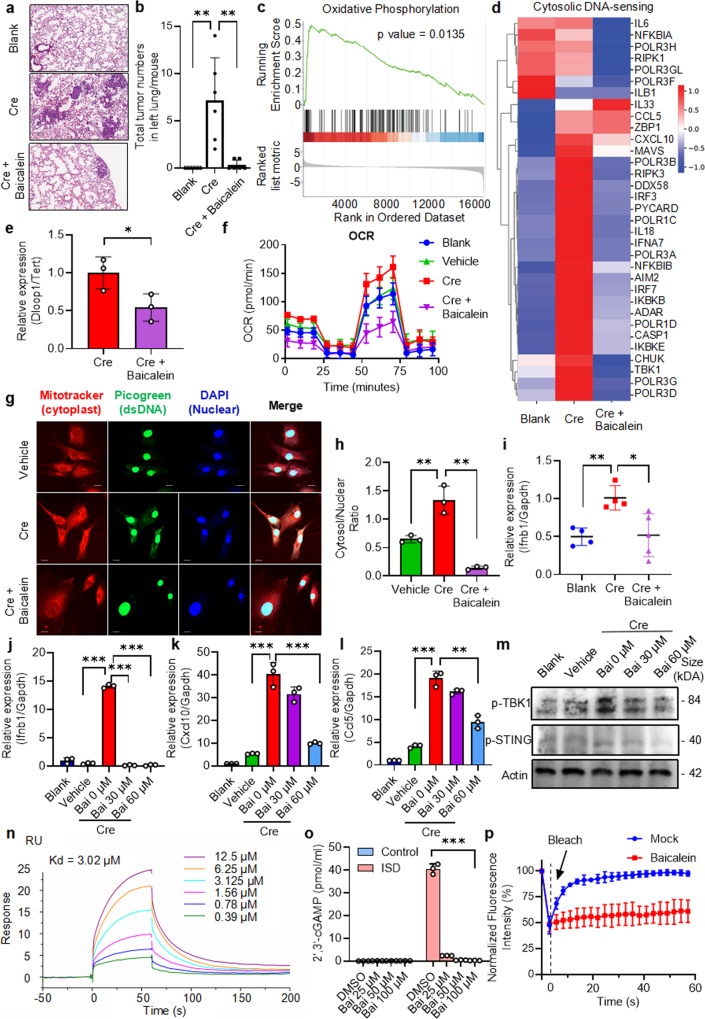
Baicalein promotes the liquid-to-solid phase transition of cGAS to suppress lung tumorigenesis. **a** Hematoxylin and eosin (H&E) staining of the section of lung tissue from mice of indicated groups. A lung adenocarcinoma (LUAD) model using conditional alleles of Kras^LSL-G12D^; p53^flox/flox^ in mice (KP mice) was established. The mice were divided into three groups, one group was treated with adenovirus expressing Cre recombinase and fed with a normal diet (Cre), and the other group was also treated with adenovirus expressing Cre recombinase and mixed with Baicalein in a normal diet (Cre+Baicalein). The third group was treated with control adenovirus and fed with a normal diet (Cre) (Blank). Scale bars left: 200 μm. The red frame indicates the magnified area, which is shown on the right side. **b** The analysis of the number of tumors formed in one lobe of the left lung in mice of the indicated groups. The symbol indicates one mouse from *n* = 6 mice per group. The data shown are representative of *n* = 3 independent experiments. Data were expressed as the mean ± SD and one-way ANOVA followed by Dunnett’s post hoc test was used for the statistical analysis. **c** Gene set enrichment analysis (GSEA) for oxidative phosphorylation pathways correlated with the differentially expressed genes (DEGs). **d** Heatmap of DEGs in the pathway of cytosolic DNA-sensing. **e** qRT-PCR measurement of mtDNA in MEF cells treated as indicated. DNA was extracted from digitonin extracts of MEF cells generated from LSL-Kras^G12D/WT^;p53^flox/flox^ mice that has been stably transduced with HBAD-Cre (Cre) left untreated or treated with Baicalein (60 μM) for 24 h. Cytosolic mtDNA was quantitated via qRT-PCR using a mitochondrial D-loop primer set. Normalization was performed as described in the Methods. **f** Oxygen consumption rate (OCR) in untreated MEF cells (Control) or MEF cells that has been stably transduced with an empty vector (Vehicle) or HBAD-Cre (Cre) in the absence of the presence of Baicalein (60 μM) for 24 h. *n* = 3. **g**, **h** Representative confocal microscopy images showing the presence of dsDNA in MEF cells that has been stably transduced with HBAD-Cre (Cre) in the absence of the presence of Baicalein (60 μM) for 24 h. Cells were stained with MitoTracker Red (red), Picogreen (green), and DAPI (blue), visualized by confocal microscopy. Scale bars = 10 μm. The quantification of the cytosolic Picogreen signal is shown in (**d**). **i** qRT-PCR measurement of *Ifnb1* transcripts in the lung of mice as in (Fig. 1a). **j**–**l** qRT-PCR measurement of transcripts of *Ifnb1* (**a**), *Cxcl10* (**b**), and *Ccl5* (**c**) in untreated MEF cells generated from LSL-Kras^G12D/WT^;p53^flox/flox^ mice (Blank), and MEF cells that has been stably transduced with an empty vector (Vehicle) or an HBAD-Cre (Cre) in the absence or presence of Baicalein at indicated concentrations for 12 h. *n* = 3. **m** Immunoblotting of indicated protein in untreated MEF cells generated from LSL-Kras^G12D/WT^;p53^flox/flox^ mice (Blank), and MEF cells that has been stably transduced with an empty vector (Vehicle) or an HBAD-Cre (Cre) in the absence or presence of Baicalein at indicated concentrations for 12 h. **n** Surface Plasmon Resonance (SPR) assay showing the binding of Baicalein with cGAS (Kd = 3.02 μM). **o** An ELISA assay of the production of cGAMP by purified SUMO-cGAS in the absence or presence of ISD left untreated or treated with increasing dose of Baicalein. **p** FRAP assay of mEGFP-cGAS in Hela cells. The intensity was normalized with the pre-bleached as 100%. Graphical data were mean ± SD. Statistical analyses were done using unpaired Student’s *t*-test (**d**, **f**) or one-way ANOVA followed by Dunnett’s post hoc test (**a**). *p < 0.05; **p < 0.01

We then performed transcriptomics analysis of lung tissues from mice in three indicated groups. Intriguingly, Baicalein increased oxidative phosphorylation as revealed by gene set enrichment analysis (Fig. 1c and Supplementary Fig. 2a, b). The transcriptomic analysis also revealed that Kras/p53 mutation induced the activation of the cytosolic DNA sensing pathway, including a variety of downstream factors, which were reversed by Baicalein (Fig. 1d). Collectively, the restoration of mitochondria function and inhibition of cytosolic DNA sensing pathway may be responsible for Baicalein-mediated abrogation of lung tumorigenesis.

Next, we examined whether mitochondrial dysfunction is involved in tumorigenesis by genetic ablation of Kras/p53 in mouse embryonic fibroblast (MEF) cells. The introduction of Cre recombinase into MEF cells promoted the transcription of the Kras^G12D^ gene and deleted the expression of p53 (Supplementary Fig. 3a). It significantly increased the level of mt-16S and mt-Dloop1 in the cytosol of MEF cells (Supplementary Fig. 3b), indicating that the genetic ablation of Kras and p53 may induce mtDNA release. Moreover, Cre treatment greatly improved mitochondrial function and enhanced oxygen consumption rate (OCR) in MEF cells (Supplementary Fig. 3c). The enhancements of OXPHOS are essential for the replenishment of the cellular ATP pool to accommodate increased ATP consumption in inflammation.^5^ The DNA sensor cyclic GMP-AMP synthase (cGAS) is responsible for the recognition of released mtDNA and subsequent activation of type I interferon responses.^6^ Kras/p53 mutation markedly induced the transcripts of *Cxcl10* and *Ccl5* (Supplementary Fig. 3d, e) as well as phosphorylation of TBK1, IRF3, and STING (Supplementary Fig. 3f). Meanwhile, we found that cGAS was highly expressed in clinical lung cancer tissue (Supplementary Fig. 3g), indicating that cGAS and its downstream type I interferon signal may play a role in the development of lung cancer.

Previous studies proved that Baicalein has a protective effect on mitochondria.^7^ We further found that Baicalein markedly reversed the rise of OCR caused by Cre treatment and significantly reduced the release of mtDNA into the cytosol (Fig. 1e–h and Supplementary Fig. 4a). Immunohistochemistry staining of dsDNA demonstrated that accumulation of cytosolic DNA was inhibited by Baicalein in lung tissues of indicated mice (Supplementary Fig. 4b, c). Our data suggest that Baicalein may maintain the normal function of mitochondria and impede the release of mtDNA into the cytosol.

Then we examined whether Baicalein directly inhibits cGAS-STING pathway activation. Baicalein significantly inhibited immunostimulatory DNA (ISD)-induced expression of *Ifnb1* and *Cxcl10* in a dose-dependent manner (Supplementary Fig. 5a, b). Moreover, Baicalein inhibited Cre-mediated induction of type I IFN response, including *Ifnb1*, *Cxcl10*, and *Ccl5* in MEF cells (Fig. 1j–l) as well as phosphorylation of STING and TBK1 (Fig. 1m). Kras/p53 mutation caused the synthesis of *Ifnb1* in the lung tissue of mice, which was markedly reduced by Baicalein (Fig. 1i). TBK1 activation has been revealed to be critical for the inflammation-driven tumorigenesis.^8^ Immunohistochemistry staining revealed that Baicalein profoundly inhibited Cre-mediated phosphorylation of TBK1 in the lung tissue of mice (Supplementary Fig. 5c, d). These results indicate that Baicalein may inhibit cGAS-STING signaling pathway activation from suppressing inflammation-driven carcinogenesis.

To examine the mechanism, we detected the interaction of Baicalein with cGAS. Surface plasmon resonance assay revealed a direct interaction of Baicalein with recombinant cGAS with a dissociation constant *K*_d_ of 3.02 μM (Fig. 1n). An immunoprecipitation with biotin-labeled Baicalein also demonstrated a direct interaction of Baicalein with recombinant cGAS (Supplementary Fig. 6a). An immunoprecipitation of biotin-ISD demonstrated that the presence of Baicalein impaired the interaction of ISD with cGAS (Supplementary Fig. 6b). Furthermore, an ELISA detection of the amount of cGAMP showed that Baicalein inhibited the enzymatic activity of cGAS in a dose-dependent manner (Fig. 1o).

Phase separation has been demonstrated to be critical in the regulation of cGAS activation.^9^ Immunofluorescence assay revealed that ISD stimulation resulted in the liquid phase separation of GFP-cGAS in HeLa cells (Supplementary Fig. 6c). FRAP assay showed that Baicalein led to a failed recovery of quenched fluorescent signaling (Fig. 1p and Supplementary Fig. 6c). Moreover, Baicalein markedly reduced LLPS of endogenous cGAS caused by the mutation of Kras/p53 (Supplementary Fig. 6d, e), which may partly due to the inhibitory effect of Baicalein on Kras/p53 mutation-triggered mtDNA release (Fig. 1g, h). Notably, Baicalein treatment led to a failed recovery of quenched fluorescent signaling in MEF cells (Supplementary Fig. 6d, f). These results suggested that Baicalein may induce the liquid-to-solid transition of cGAS to impede its activation.

A molecular docking assay of the complex of Baicalein with cGAS indicated that Y248, R376, L377, S378, F379, E383, Y436, N482, I485, and F486 are responsible for the binding of cGAS to Baicalein (Supplementary Fig. 7a). An immunoprecipitation with biotin-Baicalein revealed that the deletion of RLSF (∆376–379) motif completely impaired the interaction of Baicalein with cGAS (Supplementary Fig. 7b). The deletion of RLSF (∆376–379) markedly reduced cGAS-induced STING activation (Supplementary Fig. 7c). Importantly, Baicalein did not further inhibit the activation of cGAS^∆376–379^ (Supplementary Fig. 7c), indicating that Baicalein may target amino acids 376–379 of cGAS to mediate its inhibitory effect. The C-terminal lobe of cGAS contains a conserved zinc-ion-binding module that can promote DNA-induced phase separation and lead to stronger enzymatic activity.^9,10^ Therefore, it’s highly possible that the deletion of amino acids 376–379 may result in a conformational change of the zinc finger structure and thereby impair the LLPS of cGAS. We further purified SUMO-tagged cGAS and cGAS^∆376–379^ (Supplementary Fig. 7d). SUMO-cGAS^∆376–379^ showed much attenuated enzymatic activity in the synthesis of cGAMP in comparison to SUMO-cGAS (Supplementary Fig. 7e). However, the inhibitory effect of Baicalein on the enzymatic activity of SUMO-cGAS was not observed for SUMO-cGAS^∆376–379^ (Supplementary Fig. 7e). By constructing mEGFP-cGAS with 376–379 amino acid residue mutation (mEGFP-cGAS^∆376–379^) and observing its distribution in HeLa cells, we found that the mutated cGAS protein did not form liquid droplets and undergo liquid phase separation in the cytosol (Supplementary Fig. 7f). In conclusion, the amino acid residues 376–379 are the key sites responsible for the liquid phase separation of cGAS and its activation, which could be directly targeted by Baicalein.

In summary, our work established a critical role of cGAS-mediated sensing of mtDNA in driving tumorigenesis caused by genetic ablation of Kras and p53. Importantly, Baicalein was identified as an appealing therapeutic agent for the early intervention of lung cancer by playing dual roles in suppressing mtDNA release and inhibiting cGAS activation (Supplementary Fig. 8, Diagram). Moreover, Baicalein was identified as a novel cGAS inhibitor by promoting its liquid-to-solid phase transition, providing a powerful tool for the interventions of diseases associated with cGAS overactivation.

## Supplementary information

Supplementary

Figure S1

Figure S2

Figure S3

Figure S4

Figure S5

Figure S6

Figure S7

Figure S8

## Data Availability

The data sets used for the current study are available from the corresponding author upon reasonable request.
