# Supplementary material for: Promotion of liquid-to-solid phase transition of cGAS by Baicalein suppresses lung tumorigenesis
Source: Signal Transduct Target Ther. 2023 Mar 22;8:133. doi: 10.1038/s41392-023-01326-6 (PMC10030660; doi:10.1038/s41392-023-01326-6)
Supplement: Supplementary file 1 — Supplementary [file 41392_2023_1326_MOESM1_ESM.docx]

**Promotion of liquid-to-solid phase transition of cGAS by Baicalein**

**suppresses lung tumorigenesis**

Haipeng Liu^1,2,#^, Tiansheng Zheng^2,#^, Yifan Hong^3,#^, Yajuan Cao^1^, Qing Xia^2,3^, Chengge Qin^3,4^, Russel J. Reiter^5^, Yidong Bai^5^, Baigenzhin Abay^6^, Lihong Fan^2,3*^

^1^ Clinical Translational Research Center, Shanghai Pulmonary Hospital, Tongji University School of Medicine, Shanghai 200433, China.

^2^ Department of Respiratory Medicine, Shanghai Tenth People’s Hospital, Tongji University School of Medicine, Shanghai 200072, China.

^3^ Institute of Energy Metabolism and Health, Shanghai Tenth People’s Hospital, Tongji University School of Medicine, Shanghai 200072, China.

^4^ Medical School of Nantong University, Nantong, Jiangsu 22601, China.

^5^ Department of Cell Systems and Anatomy, University of Texas Health San Antonio, San Antonio, Texas 78229, USA

^6^ National Scientific Medical Research Centre 010009, Astana, Kazakhstan

# These authors contributed equally to this study.

* To whom correspondence should be addressed, E-mail: Lihong Fan (fanlih@ aliyun.com).

**Materials and methods**

**Antibodies and Reagents**

The following antibodies were obtained commercially: rabbit anti-TBK1 (T55145, Abmart); mouse anti-Phospho-TBK1(Try179) (M011924, Abmart); rabbit anti-IRF3 (T55779, Abmart); rabbit anti-Phospho-IRF3 (S386) (T56575, Abmart);rabbit anti-TMEM173/STING (TD12090, Abmart);rabbit anti-TMEM173/STING (TD12090, Abmart); rabbit anti-Phospho-STAT1 (S727) (T55702, Abmart); rabbit anti-Phospho-TMEM173/STING (Ser366) (TA7416, Abmart); mouse anti-α-tubulin (DM1A) (CST) and rabbit anti-β-Actin (D6A8) (HRP Conjugate) (6C5) (12620, CST). Picogreen dsDNA Quantitation Reagent (12641ES01, Yeasen); MitoTracker® Red CMXRos (40741ES50, Yeasen); 4’,6-Diamidino-2-phenylindole (DAPI, 40728ES03, Yeasen). Agilent Seahorse XFp Cell Mito Stress Test Kit (103011-400, Agilent); Seahorse XF base medium (103193–100, Agilent).

**Mice**

*Kras^G12D/+^* mice were crossed with *p53^fl/fl^* mice to generate lung epithelial cell conditional Kras mutation (*Kras^G12D/+^;p53^fl/f^*). The LSL-Kras^G12D^ allele was generously provided by Dr. Tyler Jacks' laboratory and has been thoroughly described elsewhere ^1^. Mutant Kras activation was achieved via intratracheal administration of adenoviral Cre to LSL-Kras mice as previously described ^1^. In brief, the mice were anesthetized with avertin, intubated with an i.v. catheter and administered a titer 5 × 10^6^ plaque-forming units (PFUs) adenoviral Cre in total volume 62.5 μl through a micropipette inserted into the pharynx.

All mice were housed in a specific pathogen-free environment at Shanghai Tenth’s Peoples Hospital and treated in strict accordance with protocols approved by the Institutional Animal Care and Use Committee of Shanghai Tenth’s Peoples Hospital.

**Patient samples**

Paraffin-embedded tissues of lung cancer from five Kras/p53 mutation NSCLC patients were obtained. The patient protocol was approved by Shanghai Tenth people’s Hospital.

**Lung tissue preparation**

At the appropriate time points, the lungs were inflated to allow for further analysis. The animals were killed and the lungs were removed and fixed in 10% buffered formalin for 24h before embedding in paraffin. Serial mid-sagittal sections (5 μm thickness) were obtained for histological analysis. A subset of the lungs was inflated and fixed with 10% buffered formalin to allow for specific immunostaining. The antibodies used were: TBK1 (AB3786, Abmart), Ki67 (M011557, Abmart), cGAS (E5V3W) (79978, CST), and anti-ds DNA antibody (ab273137, Abcam).

**Histopathologic analysis**

All mice were euthanized for gross inspection and histopathologic examination. Xenograft tumors and mouse lungs were isolated and fixed in 4% formalin. Lung lobes were embedded in paraffin, sectioned, and stained with hematoxylin and eosin (H&E). Histologic examinations were performed by a pathologist. Tumor number was counted under microscope, and tumor area was quantified using ImageJ software by measuring pixel units.

**RNA sequencing**

Total RNA was extracted from mouse kidney, liver, lung, and spleen using TRIzol RNA extraction (Thermo Fisher Scientific). After phase separation, RNA was purified using the PureLink RNA Mini Kit (Thermo Fisher Scientific). Purified total RNA sample was treated with DNase I (TURBO DNA-freeTM kit, Ambion Inc.) to remove traces of DNA. RNA libraries were prepared using the NEBNext Ultra RNA library prep kit (Illumina) according to the manufacturer’s instructions. In brief, total RNA starting with 1 μg was poly-A selected, fragmented by random priming and then converted to cDNA using ProtoScript II reverse transcriptase. The cDNA was then end-repaired, adenylated and ligated with Illumina sequencing adaptors. Libraries from all samples were pooled and sequenced using an Illumina HiSeq 4000 with 50-bp pair-end read.

**Transcriptome Analysis**

Transcripts were used to create DEG List and normalized by trimmed mean of M values (TMM) using the calcNormFactors of edgeR^2^. Poorly expressed genes with mean transcript per million (TPM) of less than 1 were removed from downstream analysis. Differential analysis among three groups was performed by using the limma package^3^, and each group contained three biological replicates. Finally, Gene Set Enrichment Analysis (GSEA) was conducted using the GSEA software downloaded from the Broad Institute website (https://www.gsea-msigdb.org/gsea/index.jsp). Mouse genes were converted to human homologs using biomaRT ^4^ before evaluating enrichment of gene sets. Enrichment plots and heat maps were generated in R Studio.

**MEF Cell Isolation**

*Kras^G12D/+^;p53^fl/fl^* mouse embryonic fibroblasts were isolated at embryonic d13.5 (E13.5) and placed on ice-cold PBS, 1× Ca^2+^, Mg^2+^ (137 mM NaCl, 2.7 mM KCl, 10 mM Na_2_HPO_4_, 1.8 mM KH_2_PO_4_, 1 mM CaCl_2_, 0.5 mM MgCl_2_). Embryos were minced after removing their liver, spleen, and intestine and then digested with 0.25% Trypsin-EDTA (C0204; Beyotime). Trypsin-EDTA incubation was stopped with complete media [high-glucose DMEM (Corning)]; supplemented with 10% heat-inactivated FBS (Gibco)], 50 U/ml penicillin/streptomycin (Corning); washed with PBS; plated in complete media; and incubated at 37°C in 5% CO2. Medium was replaced every 2 days. MEFs were frozen at Passage 1 in complete media, supplemented with 90% FBS and 10% DMSO. After thawing, at Passage 3, MEF cells were dissociated from plasticware using 0.05% Trypsin-EDTA and seeded into 24-well plates for transfection. In this study, MEF cells were transfected at Passage 4.

**Cell culture**

HeLa cell (ATCC, CCL-2.2) were cultured in Roswell Park Memorial Institute 1640 medium (RPMI 1640, Sigma-Aldrich) supplemented with 10% (v/v) heat-inactivated fetal bovine serum (FBS, Gibco). Human embryonic kidney epithelial cells (HEK293T; ATCC CRL-11268) and MEF cells, were cultured in Dulbecco’s Modified Eagle’s Medium (DMEM; Gibco) supplemented with 10% (v/v) heat-inactivated fetal bovine serum (FBS; Gibco), 1% (v/v) penicillin–streptomycin, 1 mM of sodium pyruvate, 2 mM of L-glutamine, 10 mM of HEPES buffer, and 50 µM of 2-mercaptoethanol (all from Gibco). Cells were maintained at 37°C in 5% CO2. All cells included were free of mycoplasma confirmed by the Look Out Mycoplasma PCR Detection Kit (MP0035, Sigma-Aldrich). MEF cells were transduced with HBAD-Cre or an empty vector for at least 16 hours using Polybrene at 5µg/ml (all from HanBio). HeLa cells were transfected with the plasmids carrying GFP-cGAS or GFP-cGAS^∆376-379^ with LipoFiter 3.0 (HanBio).

**Oxygen Consumption Analysis**

Cells were plated in XF24 plates (SeaHorse Biosciences) at 15,000 cells/well and the next day cellular O2 consumption was determined in a Seahorse Bioscience XF24 extracellular flux analyzer according to manufacturer instructions. Cells were maintained at 37 ℃ in normal growth medium without serum. On the day of the assay, culture media was replaced with Seahorse XF base medium supplemented with 1 mM sodium pyruvate, 2 mM glutamine, and 10 mM glucose (pH 7.4) and the cells were incubated at 37 ℃ (without CO_2_) for 1 h before the assay. Data was extracted and analyzed automatically by the Seahorse Wave software.

**Detection of mtDNA in Cytosolic Extracts**

Digitonin extracts from MEFs were generated as described elsewhere ^5^. *Kras*^G12D/+^;*p53*^fl/fl^ MEFs (7×10^6^) was transduced with HBAD-Cre or an empty vector for different timepoints with or without 60 µM baicalein for 24 hours subsequently were each divided into two equal aliquots, and one aliquot was resuspended in 500 µl of 50 µM NaOH and boiled for 30 minutes to solubilize DNA. 50 µl 1M Tris-HCl pH 8 was added to neutralize the pH, and these extracts served normalization controls for total mtDNA. The second aliquots were resuspended in roughly 500 µl buffer containing 150 mM NaCl, 50 mM HEPES, pH 7.4, and 15–25 µg/ml digitonin (EMD Chemicals). The homogenates were incubated end over end for 10 minutes to allow selective plasma membrane permeabilization, then centrifuged at 980 g for 3 min three times to pellet intact cells. The first pellet was saved as the ‘Pel’ fraction for western blotting. The cytosolic supernatants were transferred to fresh tubes and spun at 17 000 g for 10 min to pellet any remaining cellular debris, yielding cytosolic preps free of nuclear, mitochondrial, and ER contamination. DNA was then isolated from these pure cytosolic fractions using QIAQuick Nucleotide Removal Columns (QIAGEN). qPCR was performed on both whole cell extracts and cytosolic fractions using nuclear DNA primers (telomerase reverse transcriptase, Tert) and mtDNA primers (displacement loop region (Dloop1) and mitochondrial 16S ribosomal RNA (16S)), and the Ct values obtained for mtDNA abundance for whole cell extracts served as normalization controls for the mtDNA values obtained from the cytosolic fractions. This allowed effective standardization among samples and controlled for any variations in the total amount of mtDNA in control and TFAM-deficient samples. Using this digitonin method, no nuclear Tert DNA was detected in the cytosolic fractions, indicating nuclear lysis did not occur.

**Real-time quantitative RT-PCR**

RNA was extracted with TRIzol reagent from the indicated cells and subjected to real-time quantitative RT-PCR assays using the SYBR regent (Bio-Rad). The relative expression of the indicated genes was calculated using a standard curve method and was normalized to the expression of Gapdh. Gene-specific primer sets were listed:

Ccl5 forward, GCCCACGTCAAGGAGTATTT, reverse, CTTGAACCCACTTCTTCTCTGG;

Ifnb1 forward, CAGCCCTCTCCATCAACTATAAG, reverse, CCTTTGCACCCTCCAGTAAT;

Cxcl10 forward, TTTCTGCCTCATCCTGCTG, reverse, CAGACATCTCTGCTCATCATTCT;

Gapdh forward, AACAGCAACTCCCACTCTTC, reverse, CCTGTTGCTGTAGCCGTATT;

Dloop1 forward, AATCTACCATCCTCCGTGAAACC, reverse, TCAGTTTAGCTACCCCCAAGTTTAA;

mtDNA 16S forward, CACTGCCTGCCCAGTGA, reverse, ATACCGCGGCCGTTAAA,

Tert forward, CTAGCTCATGTGTCAAGACCCTCTT, reverse, GCCAGCACGTTTCTCTCGTT.

**Cellular** **fluorescence recovery after photobleaching (FRAP) Assays**

FRAP was performed on an inverted laser scanning confocal microscope (CarlZeiss LSM900). The 488 nm laser were used for FRAP measurements. Images were acquired with a 40× or 63× Plan-Apochromat NA1.40 oil-immersion objective under control of the Zeiss Zen software. Viral protein-DNA puncta were partially photobleached with 100% laser power. Bleaching was performed over a region of radius, and the post-bleaching images were collected from a z-stack of 4 mm every 2 s. Intensity traces were analyzed using ZEN2 software.

**Confocal microscopy assay**

Cells were stained with 3 μl/ml of PicoGreen variants (200 μM) for 30 min and washed by 1× PBS for two times. Then cells were stained with the mixture of 5μg/ml of DAPI and 100nM of Mitotracker for 30min at room temperature. For some experiments, cells were fixed in 4% PFA for 10 min at room temperature prior to PicoGreen staining. Stainings were analyzed using a CarlZeiss LSM900 confocal scanning microscope equipped with a 63× oil immersion objective. Images were analyzed using ZEN2 software.

**Molecular docking and dynamic simulation**

The crystal structures of the ligand binding domain from human cGAS (PDB number: 6EDC) were obtained from RCSB PDB database (https://www.rcsb.org/). The chemical structures of baicalein were retrieved from pubchem database (http://pubchem.ncbi.nlm.nih.gov/). The protein-ligand docking studies between cGAS and baicalein were based on these obtained compound structures and carried out with AutoDock (v4.2) packages. Potential binding sites within cGAS crystal structures were predicted based on grid energy calculation via AutoGrid program (v4.2.6-3). Conformation search and energy evaluation were completed with AutoDock program. The docked protein-ligand complexes were subjected to molecular dynamics simulation by Desmond module of Schrodinger software (https://www.schrodinger.com/Desmond/). Briefly, the optimal complex was placed in a cubic water box with a minimum distance of 10 Å between protein surface and box edges. Proper sodium and chloride ions were added to achieve physiological salt conditions with overall neutrality to 0.15 M concentration. Energy minimization was performed under OPLS-2005 force field. A totaling 100 ns of equilibration simulations were run in the NpT ensemble with parameters as follow: temperature, 300 K; pressure, 1.0135 bar; integration time step, 2 fs. Constraint of all bonds involving hydrogen atoms was achieved with SHAKE method Numerical integration of the cartesian equations of motion of a system with constraints: molecular dynamics of n-alkanes. Root mean square deviation (RMSD) average structures were extracted for interaction mode analysis.

**Synthesis of biotin-labeled baicalein**

D-Biotin (24.4 mg，0.1mmol)，TEA (triethylamine) (20.2mg，0.2 mmol)，TBTU (2-(1H-Benzotriazole-1-yl)-1,1,3,3-tetramethyluronium tetrafluoroborate) (38.5 mg，0.12 mmol)，was added to a solution of baicalein (27.02 mg 0.1 mmol) in 5mL anhydrous dichloromethane (DCM). The mixture was stirred overnight at room temperature. Thin-layer chromatography showed most of synthesize materials was consumed. The mixture was washed with water. The organic phase was concentrated and purified by thin layer chromatography eluted with DCM. The desired product was obtained and applied for further study.

**Purification of recombinant wild type SUMO-cGAS and SUMO-cGAS^∆376-379^**

The purification of wild type SUMO-tagged human cGAS has been described previously ^6^. Human cGAS^∆376-379^ cDNA was subcloned into a pET28a vector and BL21 (DE3) competent *E. coli* bacteria were then transfected with these constructs. Bacteria were grown in Luria-Bertani (LB) liquid medium to an optical density (OD) at 600 nm (OD600) of approximately 0.8. Subsequently, cells were induced with isopropyl β-D-1-thiogalactopyranoside (IPTG, 0.1 mM) overnight at 16 °C. Recombinant human SUMO-cGAS^∆376-379^ was purified from bacterial lysates using a (Ni)-chelating Sepharose Fast Flow (SFF) column (GE Healthcare, Little Chalfont, UK). The concentration of human cGAS, cGAS^∆376-379A^ protein was measured with a Pierce BCA Protein Assay Kit (Thermo Fisher Scientific).

**cGAMP quantitation assay**

The in vitro analysis of the enzymatic activity of cGAS was performed by mixing 1 μmol of SUMO-cGAS or SUMO-cGAS^∆376-379A^ with 1 μg ISD in the absence or presence of baicalein (50 μM) in the reaction buffer. The samples were centrifuged at 12 000×g for 5 min and the supernatants were applied for the detection of cGAMP by using cGAMP Enzyme Immunoassay Kit (K067-H1, Arbor Assays).

**Immunoprecipitation and western blot**

Cells were lysed using RIPA Lysis Buffer (Beyotime Biotechnology, China) supplemented with protease inhibitor cocktail (P8340, Sigma-Aldrich), 1 mM of PMSF and phosphatase inhibitor cocktail (P5726, Sigma-Aldrich). The lysates were centrifuged at 12,000 rpm for 10 min and the cellular debris was discarded. For immunoprecipitation, cell lysates were incubated with monoclonal anti-HA agarose (A2095, Sigma-Aldrich), Anti-FLAG M2 Affinity Gel (A2220, Sigma-Aldrich) or Streptavidin (Sepharose^®^ Bead Conjugate) (#3419, Cell Signaling Technology) at 4°C overnight. For immunoblotting, the protein sample lysate or precipitates were denatured in 1× sodium dodecyl sulfate (SDS) protein sample buffer at 95 °C for 8 min and then were resolved by electrophoresis through a 6% or 10% SDS-polyacrylamide gel. Separated proteins were transferred onto polyvinylidene difluoride membranes and were incubated with the prespecified antibodies at the indicated dilutions. An enhanced chemiluminescence reagent (Thermo Fisher Scientific) was applied for immunoblotting.

**Statistical analysis**

Data from independent experiments were expressed as the mean ± SD and one-way ANOVA followed by Dunnett's post hoc test or two-way ANOVA followed by Tukey’s post hoc test was performed for statistical analysis by using GraphPad Prism 8 (GraphPad, San Diego, CA). For all analyses, statistical significance was defined as *p* < 0.05.

**References**

1 Jackson, E. L. *et al.* Analysis of lung tumor initiation and progression using conditional expression of oncogenic K-ras. *Genes Dev* **15**, 3243-3248, doi:10.1101/gad.943001 (2001).

2 Robinson, M. D., McCarthy, D. J. & Smyth, G. K. edgeR: a Bioconductor package for differential expression analysis of digital gene expression data. *Bioinformatics* **26**, 139-140, doi:10.1093/bioinformatics/btp616 (2010).

3 Ritchie, M. E. *et al.* limma powers differential expression analyses for RNA-sequencing and microarray studies. *Nucleic Acids Res* **43**, e47, doi:10.1093/nar/gkv007 (2015).

4 Durinck, S., Spellman, P. T., Birney, E. & Huber, W. Mapping identifiers for the integration of genomic datasets with the R/Bioconductor package biomaRt. *Nat Protoc* **4**, 1184-1191, doi:10.1038/nprot.2009.97 (2009).

5 Holden, P. & Horton, W. A. Crude subcellular fractionation of cultured mammalian cell lines. *BMC Res Notes* **2**, 243, doi:10.1186/1756-0500-2-243 (2009).

6 Zhao, M. *et al.* CGAS is a micronucleophagy receptor for the clearance of micronuclei. *Autophagy*, 1-17, doi:10.1080/15548627.2021.1899440 (2021).

**Supplementary Figure Legends**

**
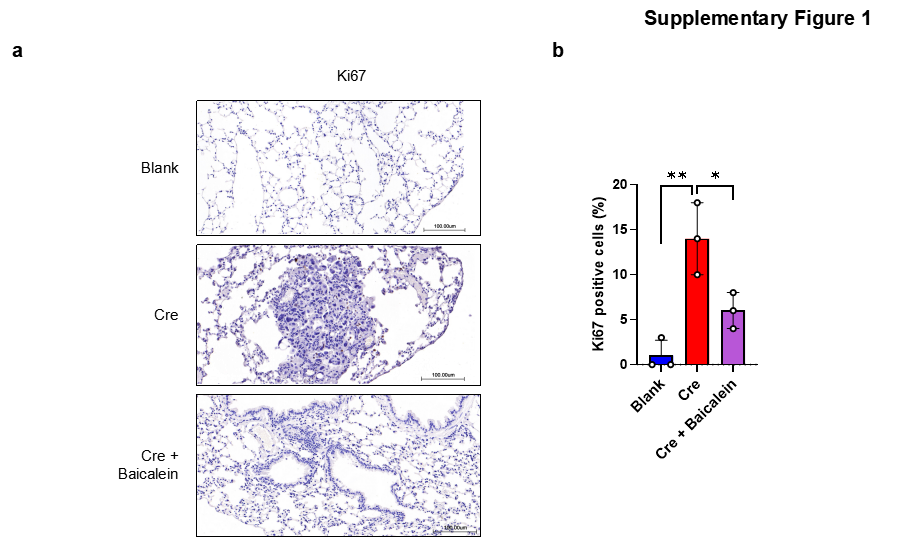
**

**Supplementary Figure 1. Baicalein suppresses Kras/p53-driven lung tumorigenesis.**

a. Representative images of immunohistochemistry staining of Ki67 in the section of lung tissue from mice of indicted groups.

b. The quantification of the percentage of Ki67 positive cells in the section of lung tissue from mice of indicted groups. Data are expressed as the mean ± SD and one-way ANOVA followed by Dunnett's post hoc test was used for the statistical analysis.

**
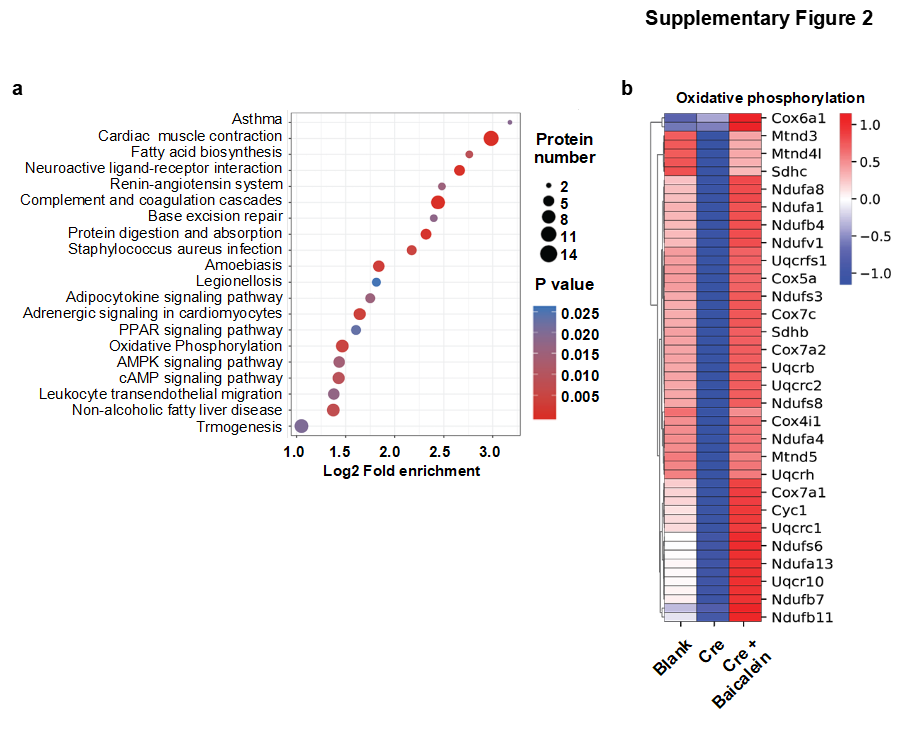
**

**Supplementary Figure 2. Baicalein protects mitochondrial function.**

a. Gene Ontology analysis of the differentially expressed genes (DEGs).

b. Heatmap of DEGs in the pathway of oxidative phosphorylation.

**
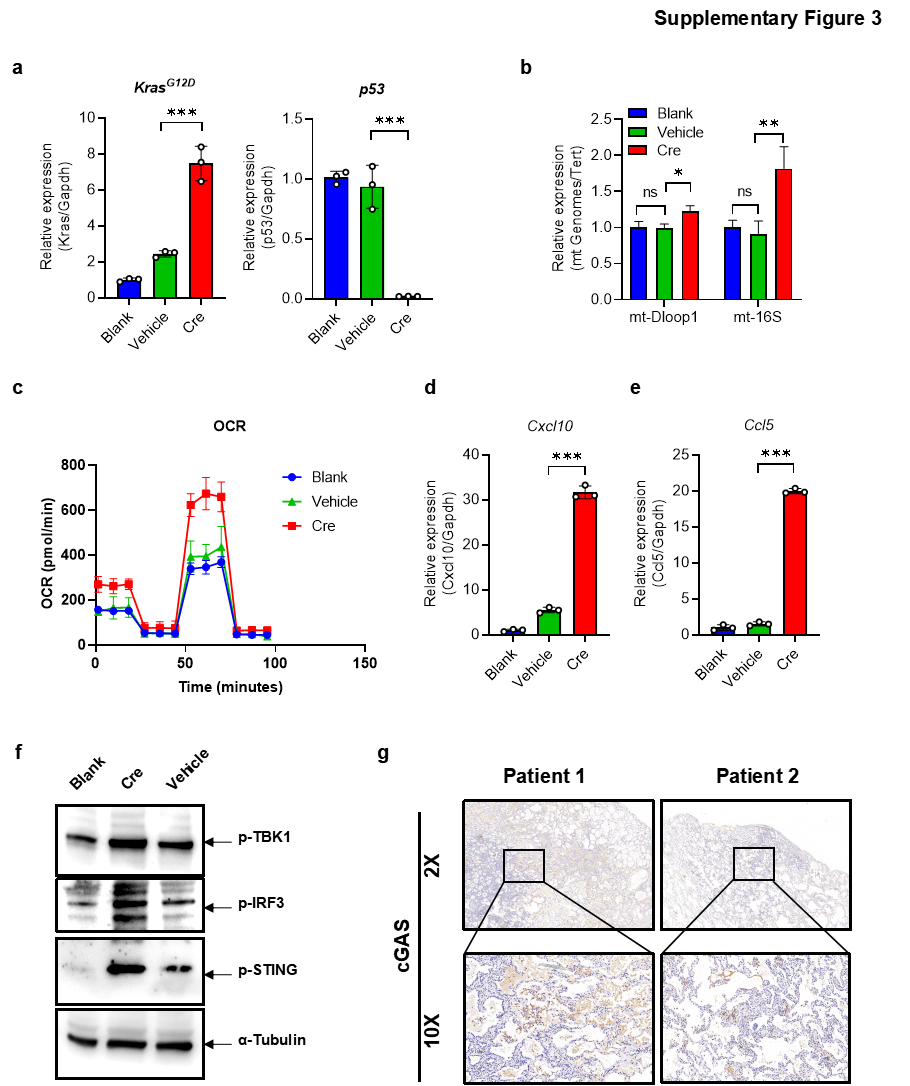
**

**Supplementary Figure 3. Kras/p53 mutation causes release of mitochondrial DNA and activation of cGAS-STING pathway.**

a. qRT-PCR measurement of transcripts of *Kars^G12D^* and *p53* in mouse embryonic fibroblast (MEF) cells generated from LSL-Kras^G12D/WT^;p53^flox/flox^ mice (control) or those that has been stably transduced with an empty vector (Vehicle) or an HBAD-Cre vector (Cre).

b. qRT-PCR measurement of cytosolic mitochondria DNA. DNA was extracted from digitonin extracts of MEF cells that has been stably transduced with HBAD-Cre or an empty vector. Cytosolic mtDNA was quantitated via qRT-PCR using a mitochondrial Dloop1 primer set or 16S. Normalization was performed as described in the Methods.

c. Oxygen consumption rate (OCR) in indicated MEF cells .

d-e. qRT-PCR measurement of *Cxcl10* (f) and *Ccl5* (g) transcripts in indicated MEF cells.

f. Immunoblotting of indicated protein in indicated MEF cells.

g. Representative immunohistochemistry staining of the expression and distribution of cGAS in tumor tissue samples of NSCLC patient with Kras/p53 mutation. Scale bar, 200 μm.

Graphical data are mean ± SD. Statistical analyses were performed using one-way ANOVA followed by Dunnett's post hoc test. *, *p* < 0.05; **, *p* < 0.01; ***, *p* < 0.001.


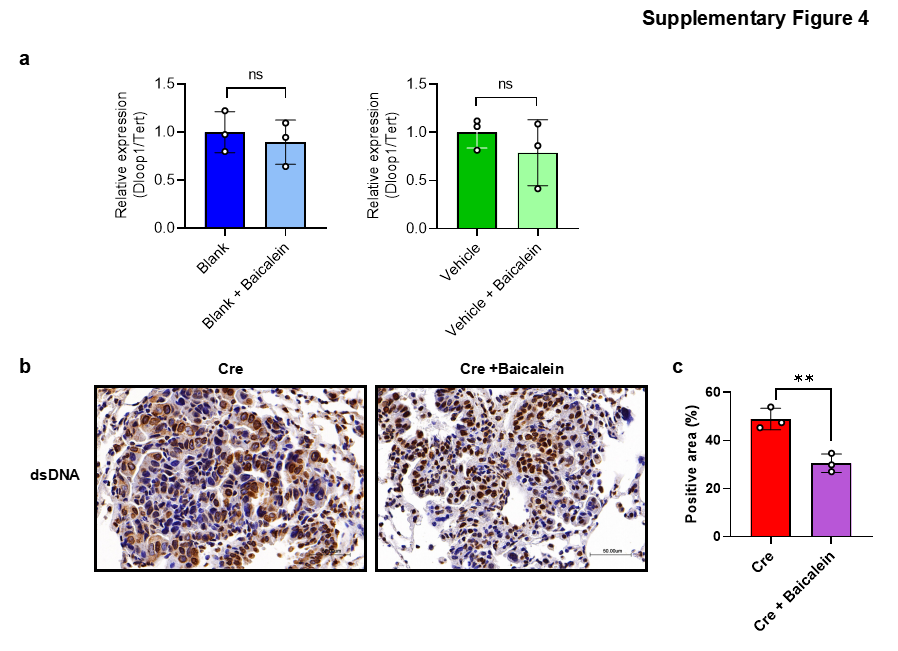


**Supplementary Figure 4. Baicalein abrogates the release of mtDNA.**

1. qRT-PCR measurement of mtDNA in MEF cells treated as indicated. DNA was extracted from digitonin extracts of MEF cells generated from LSL-Kras^G12D/WT^;p53^flox/flox^ mice that has been stably transduced with or without an empty vector (Vehicle) left untreated or treated with baicalein (60 μM) for 24 h. Cytosolic mtDNA was quantitated via qRT-PCR using a mitochondrial Dloop primer set. Normalization was performed as described in the Methods.

b-c. Representative images of immunohistochemistry staining of dsDNA in the section of lungs from mice as in (Fig 1a). Scale bar, 50 μm. The quantification data is shown in (f). n = 4 mice per group.

Graphical data are mean ± SD. Statistical analyses were done using unpaired Student’s t test (d and f) or one-way ANOVA followed by Dunnett's post hoc test (a). *, *p* < 0.05; **, *p* < 0.01

**
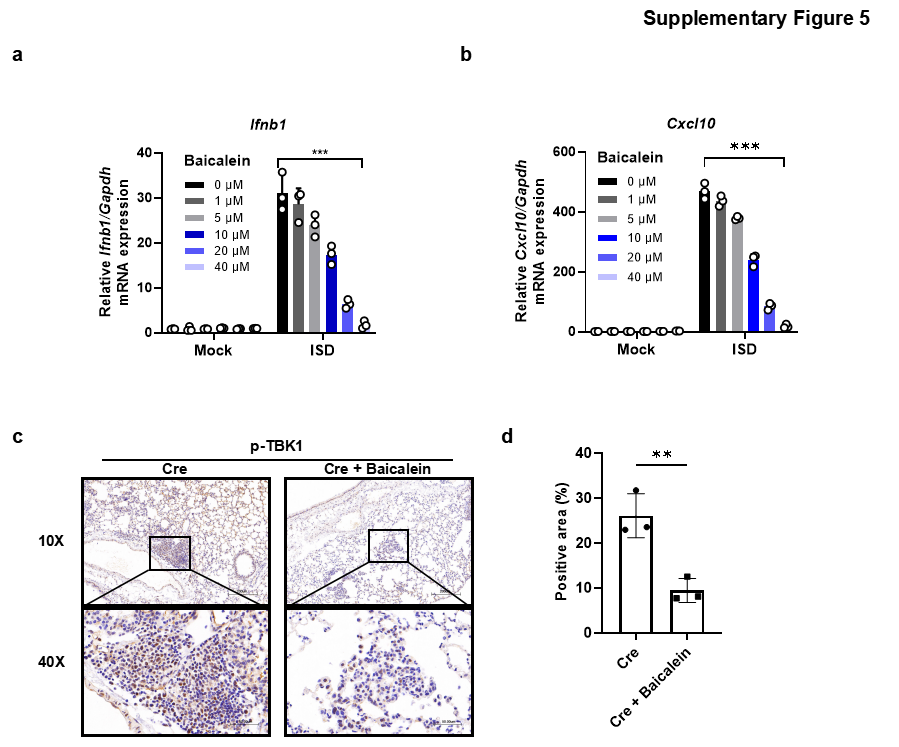
**

**Supplementary Figure 5. Baicalein inhibits cGAS-STING activation.**

a-b. qRT-PCR measurement of the transcripts of *Ifnb1* **(a)** and *Cxcl10* **(b)** in mouse peritoneal macrophages left untreated or transfected with ISD (5 μg) for 4 h in the absence or presence of increasing dose of baicalein.

c-d. Representative images showing the immunohistochemistry staining of p-TBK1 in the lung of mice as in (Fig 1a) (e). The quantification data is shown in (f). n = 4 mice per group.

Graphical data are mean ± SD. Statistical analyses were done using unpaired Student’s t test or one-way ANOVA followed by Dunnett's post hoc test. *,  *p* < 0.05; ** , *p* < 0.01; ***, *p* < 0.001.

**
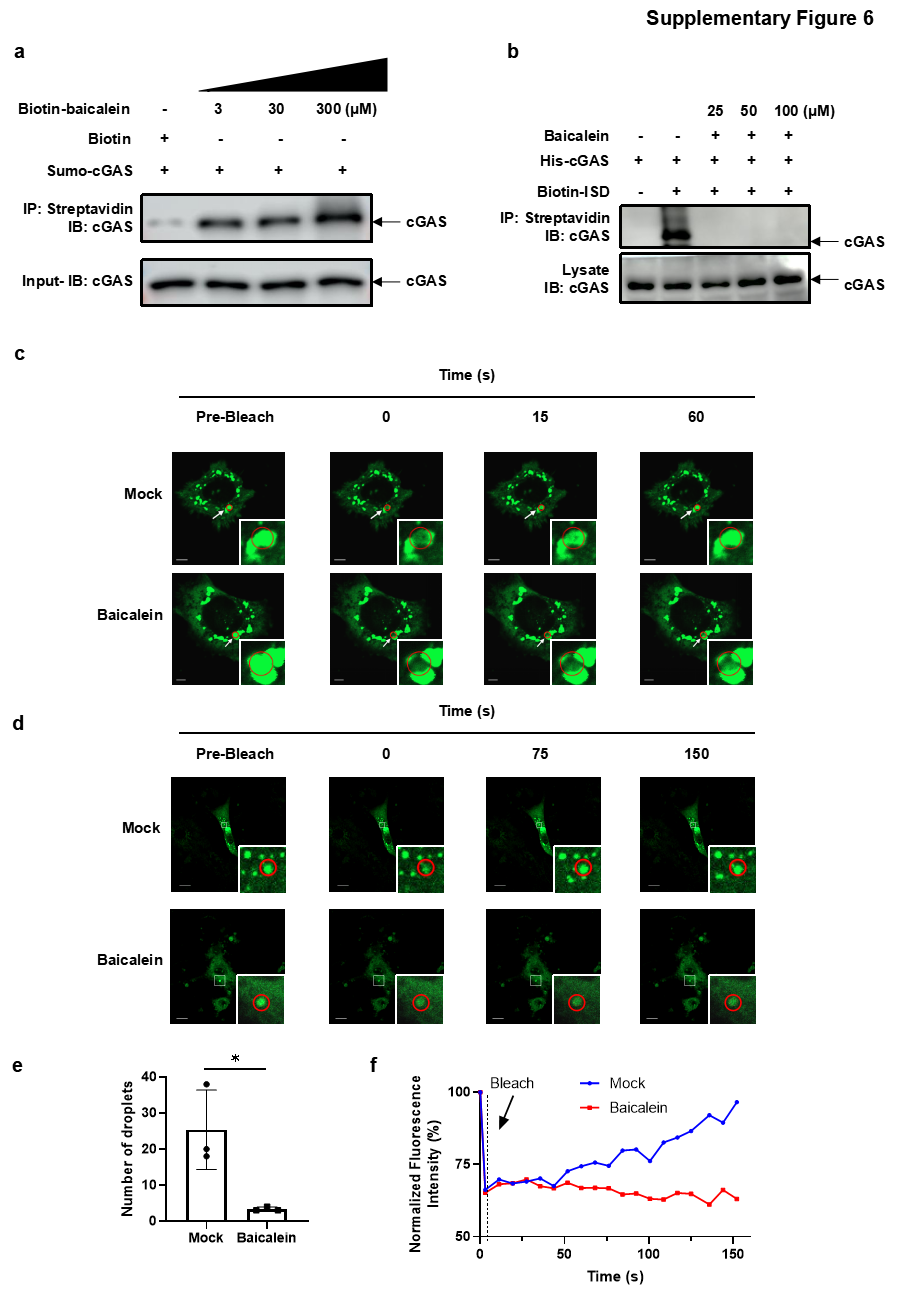
**

**Supplementary Figure 6. Baicalein directly binds cGAS and inhibits its activation by promoting the liquid-to-solid phase separation.**

a. An immunoprecipitation with streptavidin beads of SUMO-cGAS in the presence of biotin or increasing concentration of biotin-baicalein. Data are representative of n = 3 independent experiments.

b. An immunoprecipitation with streptavidin beads of His-cGAS in the absence or presence of biotin-ISD left untreated or treated with increasing dose of baicalein. IP, immunoprecipitation. IB, Immunoblotting. Data are representative of n = 3 independent experiments.

c. FRAP assay of mEGFP-cGAS in Hela cells. The fluorescence intensities of each area marked by red circles were plotted versus time.

d. FRAP assay of mEGFP-cGAS in MEF cells that have been stably transduced with HBAD-Cre left untreated or treated with baicalein (60 μM) for 24 h. The fluorescence intensities of each area marked by white rectangle were plotted versus time.

e. Droplet number in MEF cells. n = 3 independent experiments with analyzing at least 50 cells in each experiment.

f. FRAP assay of mEGFP-cGAS in MEF cells. The intensity was normalized with the pre-bleached as 100%.

**
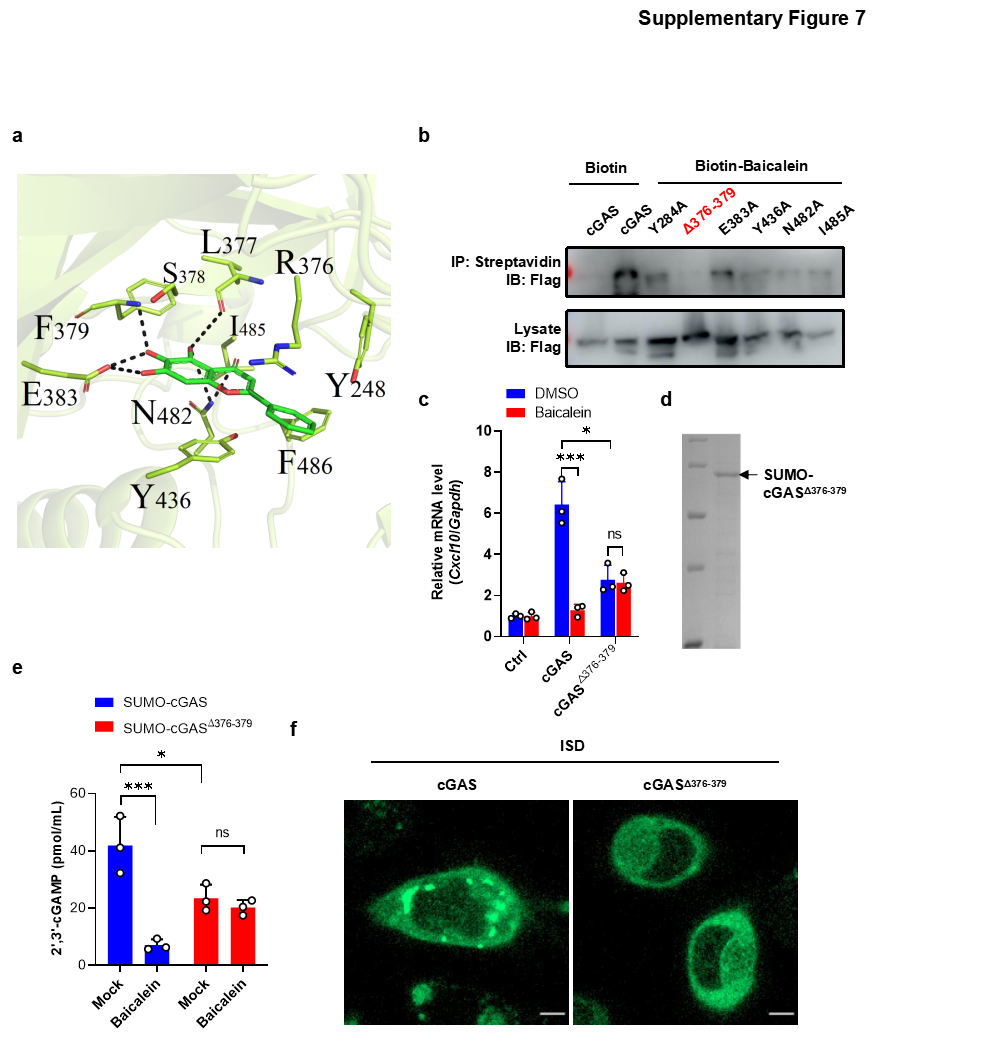
**

**Supplementary Figure 7. RLSF motif is required for baicalein-mediated inhibition of cGAS.**

a. Molecular docking showing the interaction between baicalein and cGAS. Baicalein (cyan) is docked opposing to cGAS. Ten amino acids (red) were located. The distance between the Baicalein and the cGAS in our current model is 3.7Å.

b. An immunoprecipitation with streptavidin beads of WT cGAS and corresponding cGAS mutants in the presence of biotin or biotin-baicalein.

c. qRT-PCR measurement of the abundance of *CXCL10* transcripts in STING stable HEK293 cells transfected with control vector (Ctrl), cGAS or cGAS^(∆376-379)^ (∆376-379) in the absence or presence of baicalein.

d. SDS-PAGE analysis showing the purified SUMO-cGAS^(∆376-379)^.

e. An ELISA assay showing the enzymatic activities of purified SUMO-cGAS and SUMO-cGAS^(∆376-379)^  in the absence or presence of baicalein (50 μM) by measuring the abundance of cGAMP.

f. Representative images showing the distribution of transfected mEGFP-cGAS or mEGFP-cGAS^(∆376-379)^ with ISD for 4 hours in Hela cells.

**
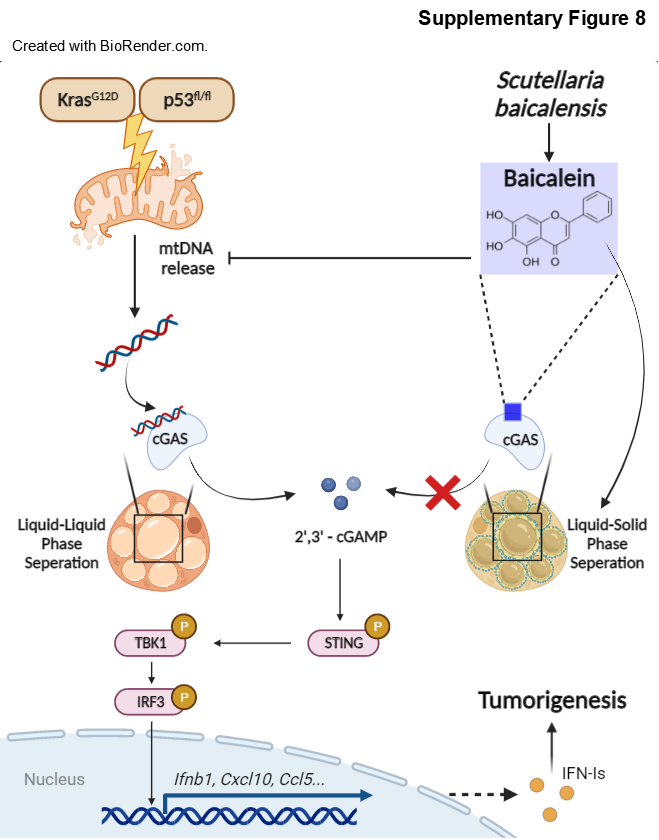
**

**Supplementary Figure 8. Diagram showing that baicalein promotes liquid-to-solid phase transition of cGAS to suppress lung tumorigenesis.**

Our work demonstrated that Kras/p53 mutation induces mitochondria dysfunction and the release of mtDNA into the cytosol. MtDNA is then engaged by the cytosolic DNA sensor cGAS and triggers type I IFN responses, which finally drives lung cancer tumorigenesis. Notably, the liquid-liquid phase separation of cGAS-DNA is required to enable DNA sensing inducing tumorigenesis. Intriguingly, baicalein, extracted from traditional Chinese medicine, abrogates the release of mtDNA by preventing mitochondrial dysfunction and binds cGAS to promote the liquid-to-solid phase separation, both of which are critical for the termination of cGAS-STING activation. Our work thereby establishes a critical role of cGAS-STING activation in lung carcinogenesis and identifies baicalein as a powerful agent in the prevention and treatment of lung cancer.
