# Supplementary figures and images for: Promotion of liquid-to-solid phase transition of cGAS by Baicalein suppresses lung tumorigenesis
Source: Signal Transduct Target Ther. 2023 Mar 22;8:133. doi: 10.1038/s41392-023-01326-6 (PMC10030660; doi:10.1038/s41392-023-01326-6)

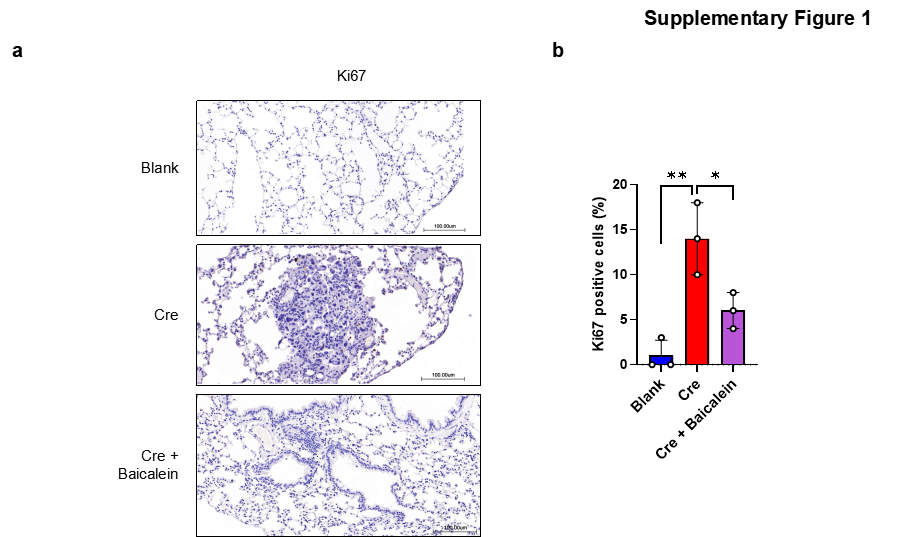

Supplement: Supplementary file 2 — Figure S1 [file 41392_2023_1326_MOESM2_ESM.png]

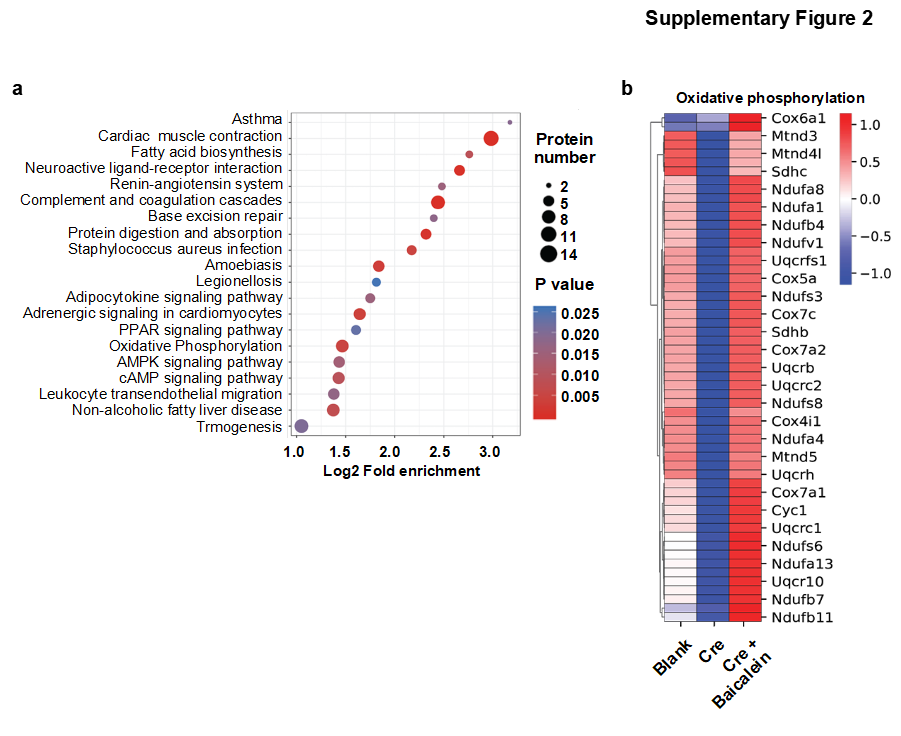

Supplement: Supplementary file 3 — Figure S2 [file 41392_2023_1326_MOESM3_ESM.png]

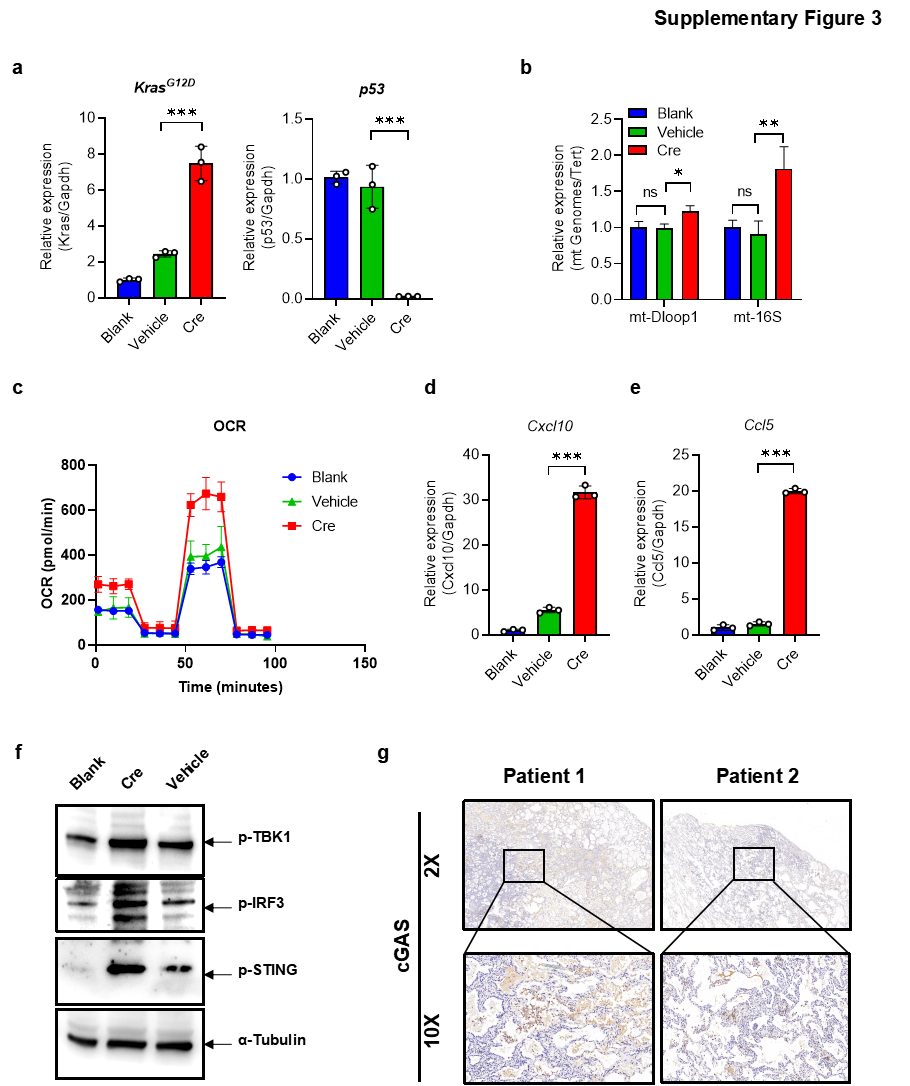

Supplement: Supplementary file 4 — Figure S3 [file 41392_2023_1326_MOESM4_ESM.png]

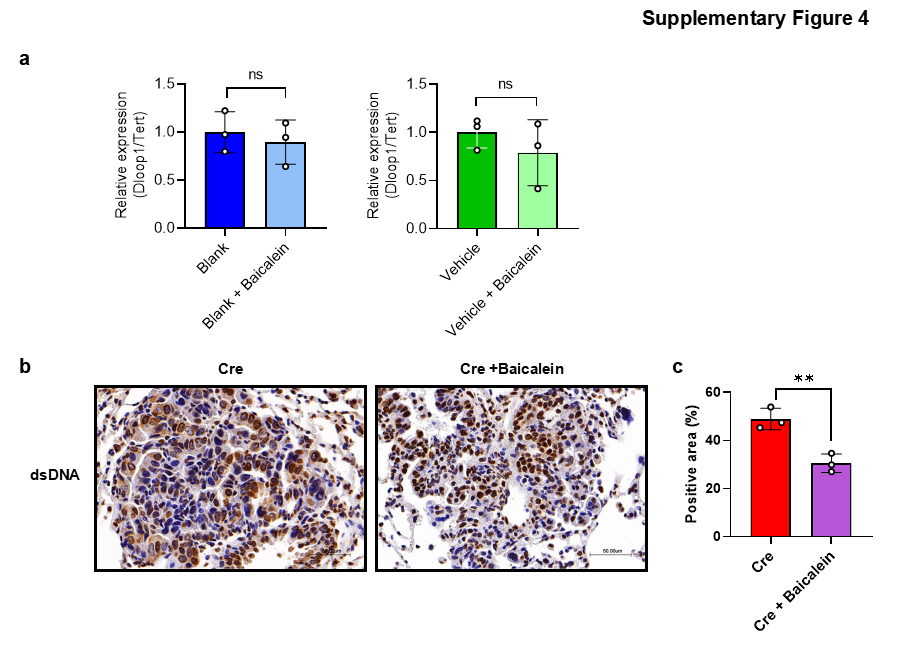

Supplement: Supplementary file 5 — Figure S4 [file 41392_2023_1326_MOESM5_ESM.png]

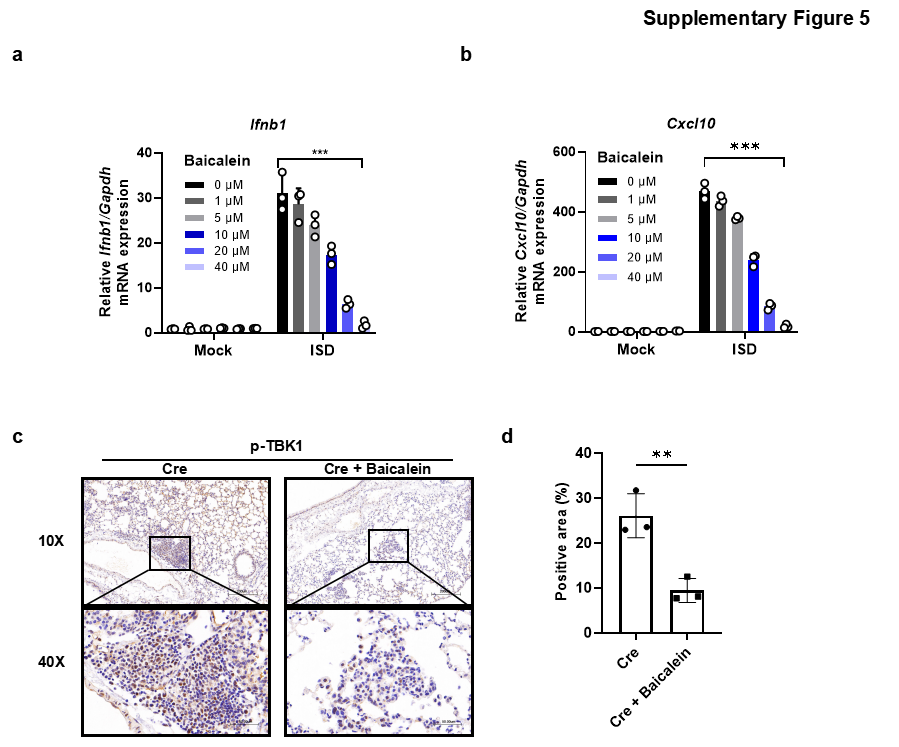

Supplement: Supplementary file 6 — Figure S5 [file 41392_2023_1326_MOESM6_ESM.png]

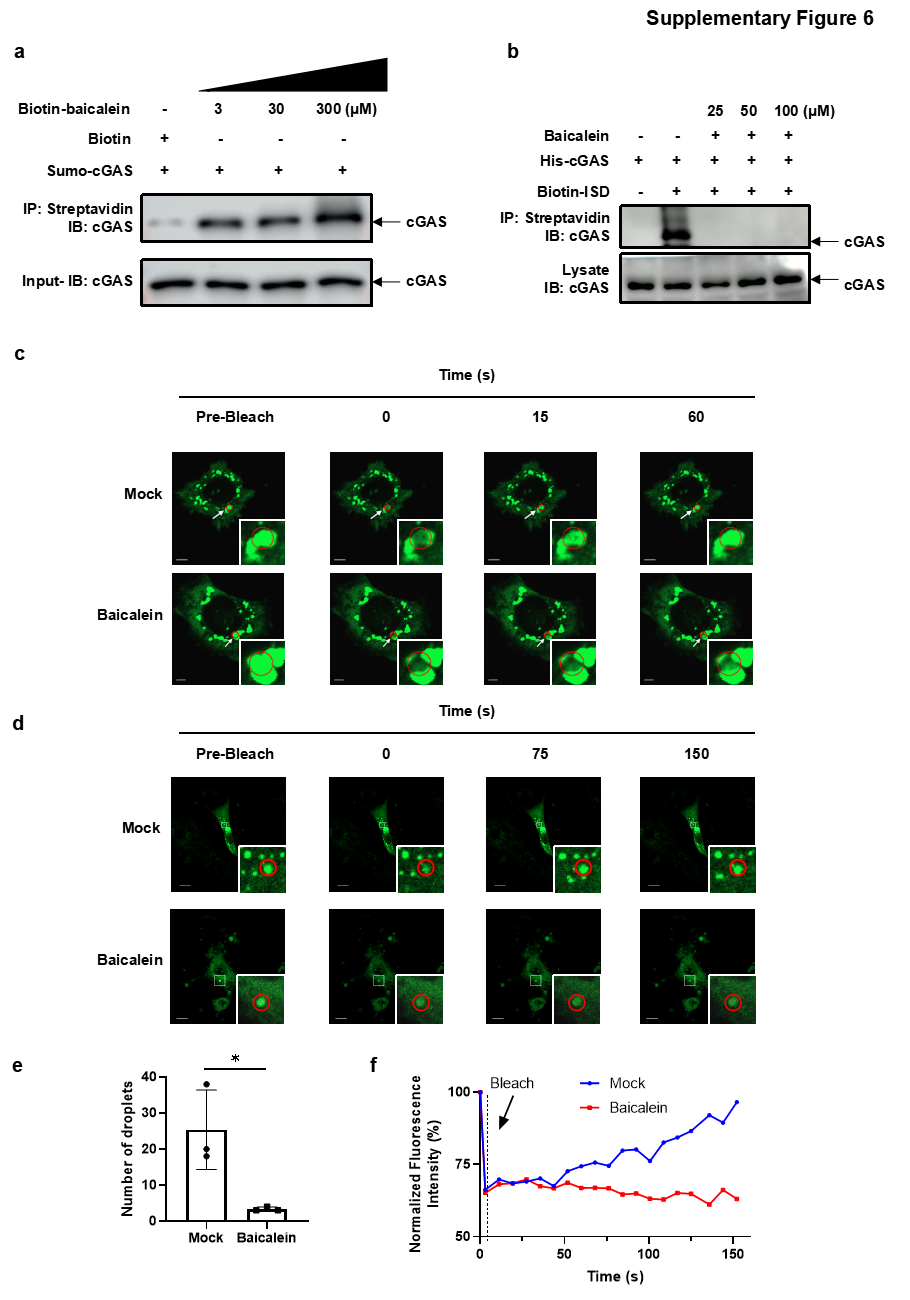

Supplement: Supplementary file 7 — Figure S6 [file 41392_2023_1326_MOESM7_ESM.png]

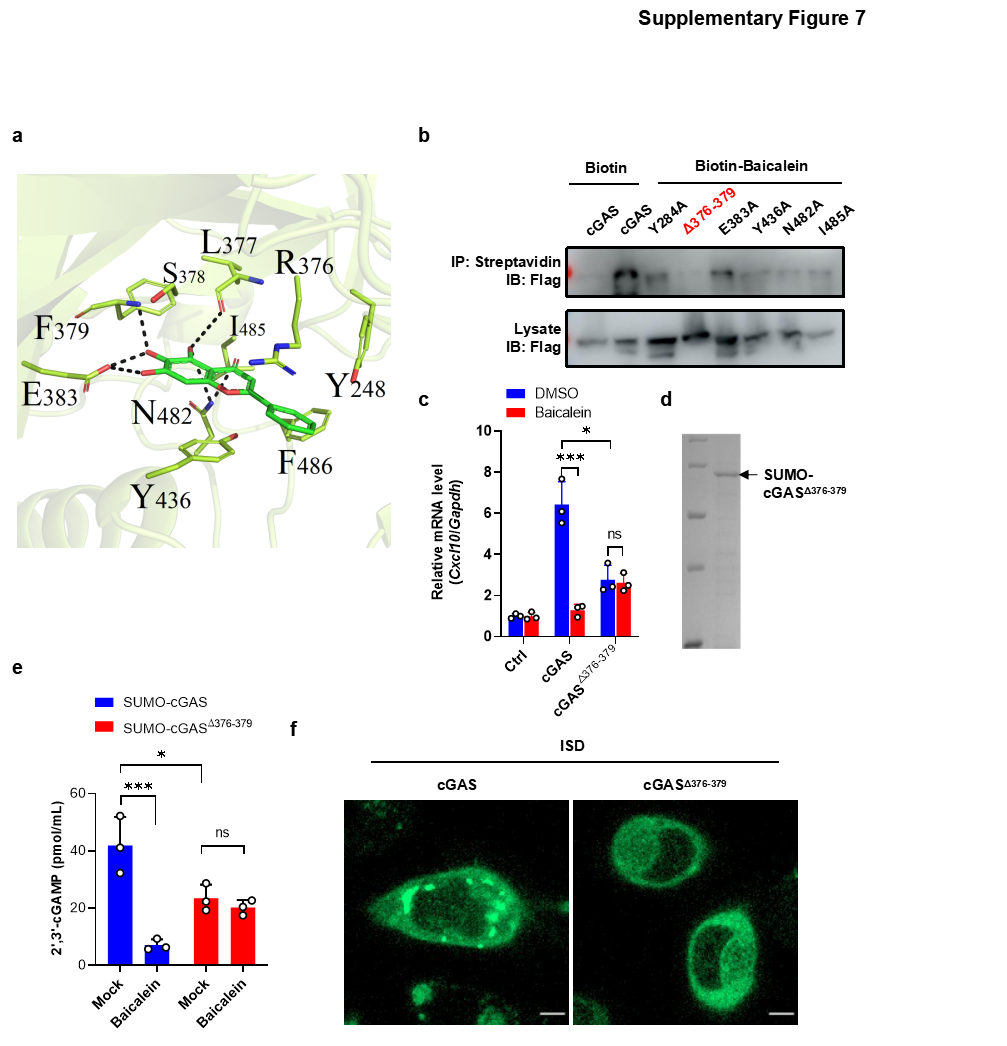

Supplement: Supplementary file 8 — Figure S7 [file 41392_2023_1326_MOESM8_ESM.png]

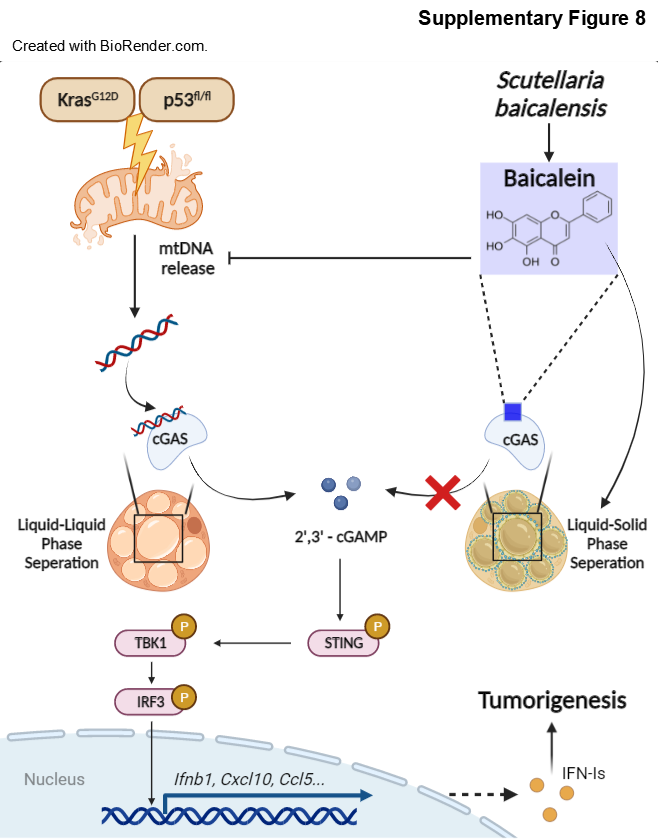

Supplement: Supplementary file 9 — Figure S8 [file 41392_2023_1326_MOESM9_ESM.png]
